# Supplementary material for: The 9-1-1 complex protects ssDNA gaps in BRCA2-deficient cancer
Source: bioRxiv. 2025 Oct 7:2025.10.07.680950. Preprint. [Version 1] doi: 10.1101/2025.10.07.680950 (PMC12632636; doi:10.1101/2025.10.07.680950)
Supplement: Supplement 2 [file NIHPP2025.10.07.680950v1-supplement-2.pdf]

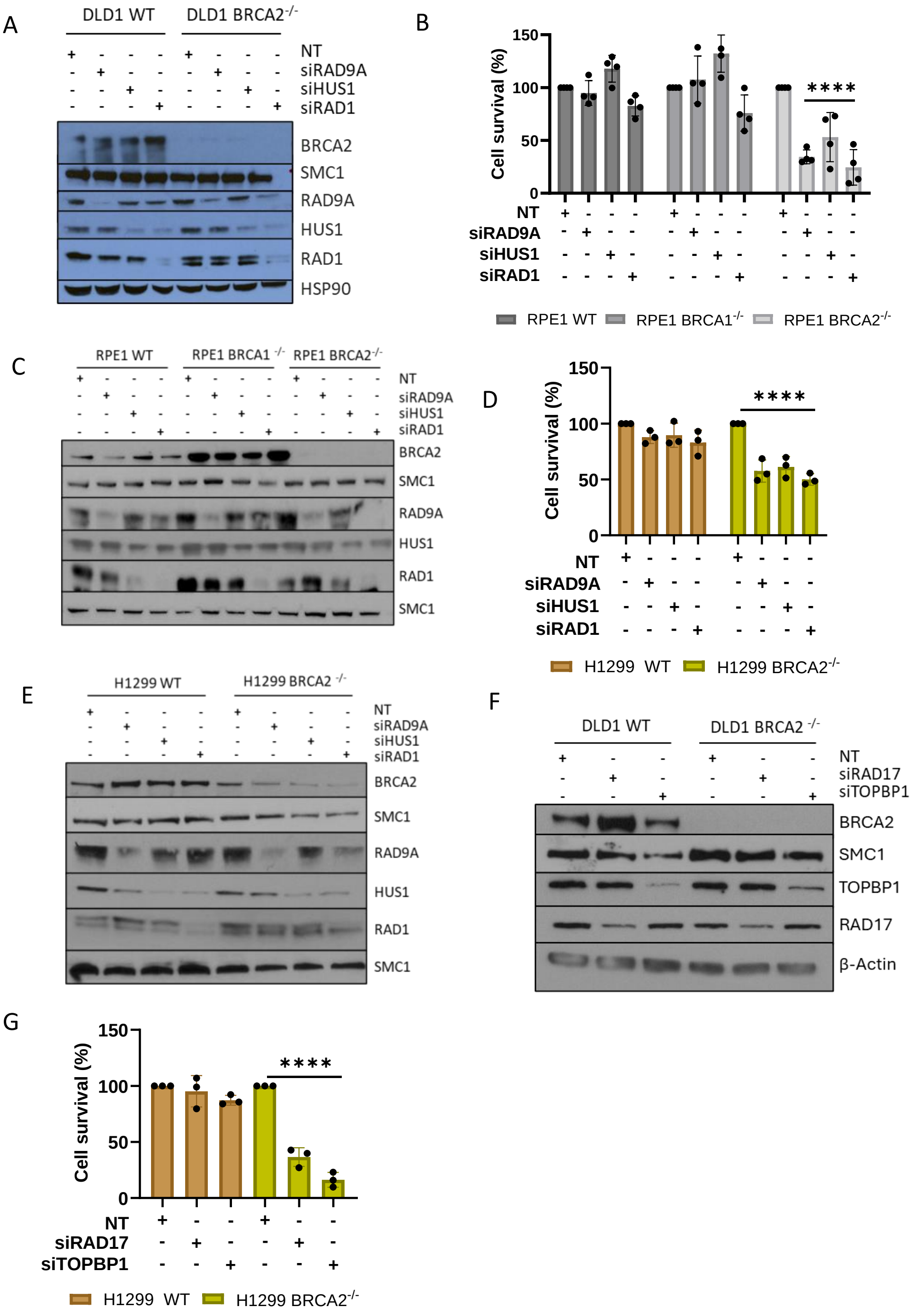

Supplementary Figure 1

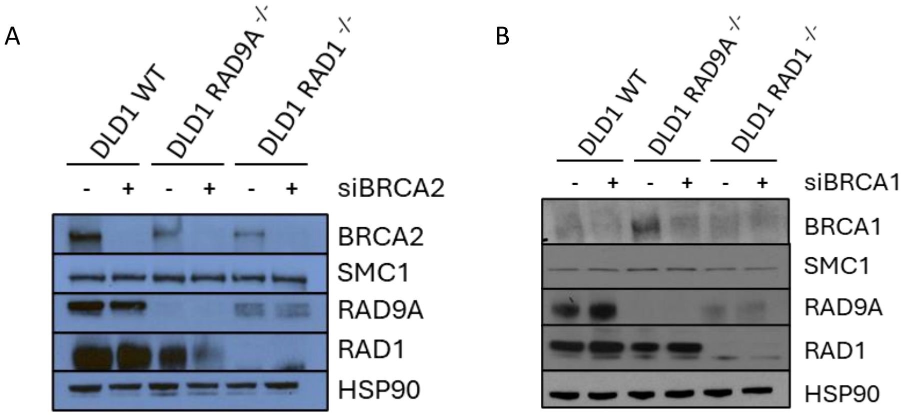

Supplementary Figure 2

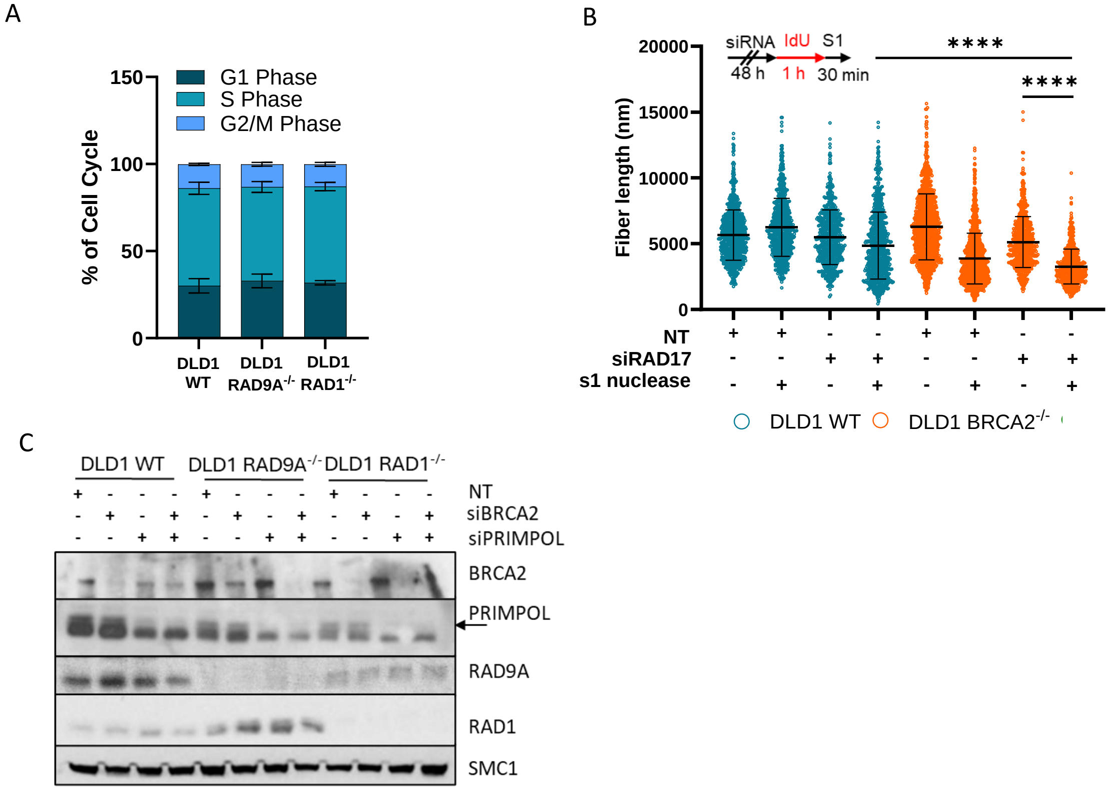

Supplementary Figure 3

A

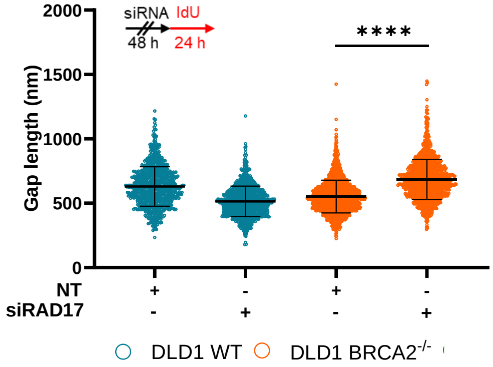

B

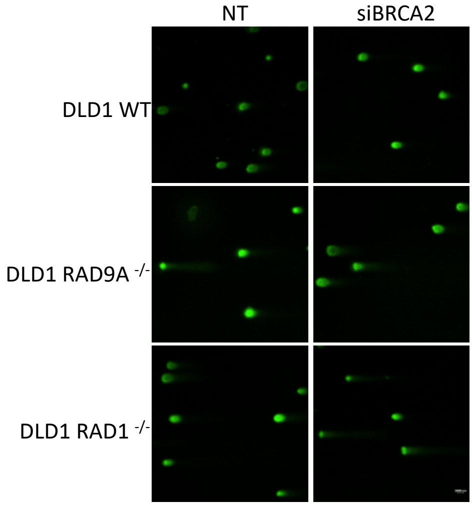

Supplementary Figure 4

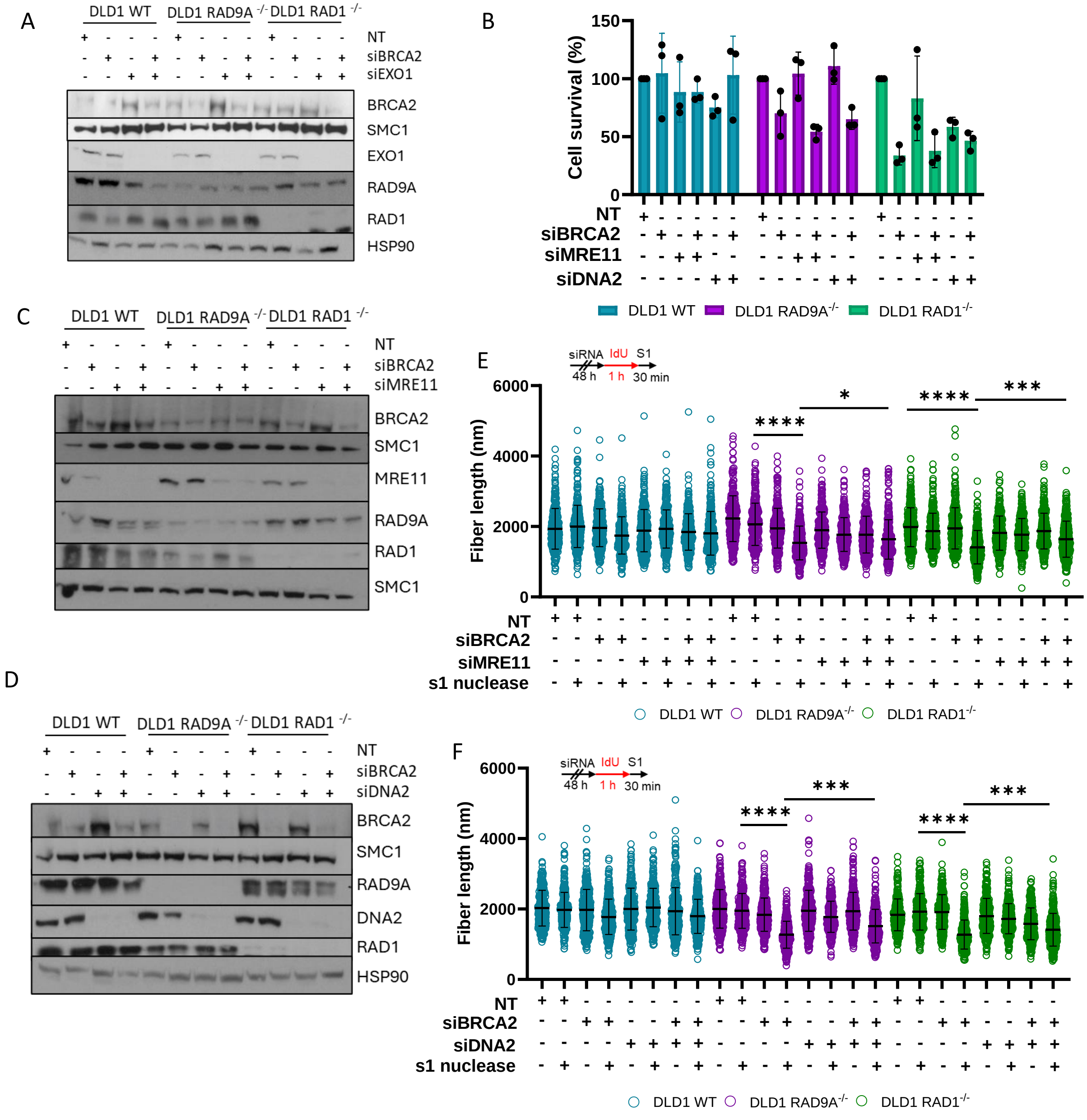

Supplementary Figure 5

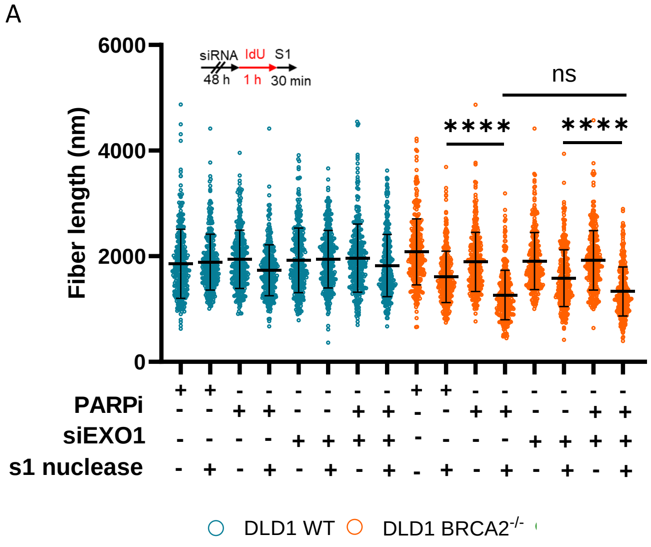

Supplementary Figure 6

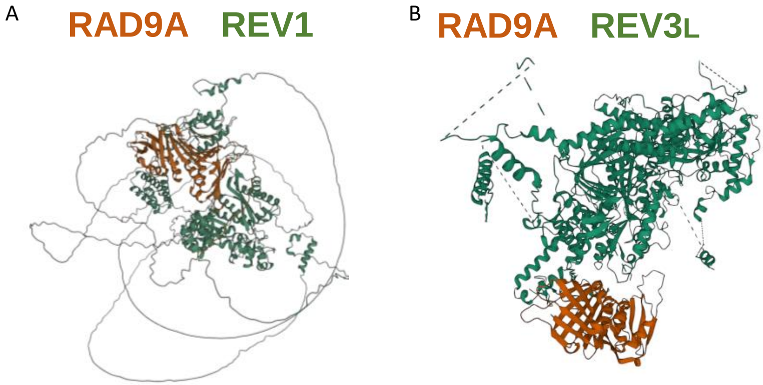

Supplementary Figure 7

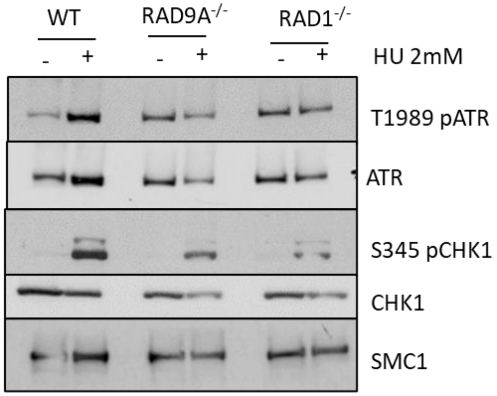

Supplementary Figure 8
